# Using video feedback to improve early father–infant interaction: A pilot study

**DOI:** 10.1177/1359104512437210

**Published:** 2013-01

**Authors:** Peter J. Lawrence, Beverley Davies, Paul G. Ramchandani

**Affiliations:** University Department of Psychiatry, Warneford Hospital, Oxford, UK

**Keywords:** Child, father, interaction, intervention, video

## Abstract

Preventive interventions with parents of infants have tended to focus on mothers. Recent research focused on fathers suggests that their involvement in interventions might enhance effectiveness. One effective approach with mothers is the brief, home-based Video-feedback Intervention to promote Positive Parenting (VIPP). This paper is a report of a pilot study of VIPP with fathers to assess its feasibility.

Five fathers were recruited from an existing longitudinal study of parents. The primary outcome was acceptability, assessed using a semi-structured questionnaire after completion of the intervention.

All fathers completed all sessions of the intervention. Fathers rated the intervention as having had a significant impact on their understanding of their child’s thoughts and feelings, and as having improved their communication and relationship with their baby. Fathers’ feedback was generally positive. The flexibility to conduct sessions at home (or at fathers’ places of work) and the flexible timing of sessions were identified as fundamental to successful delivery.

The results of this pilot study are encouraging, as VIPP with fathers was feasible. In light of the modest sample size, and the use of a non-clinical sample, the intervention must be evaluated with larger, clinical samples to evaluate its efficacy with fathers.

## Background

There is accumulating evidence that interventions early in children’s lives can prove particularly effective in preventing the occurrence of a range of problems, including behavioural problems, emotional disorders and attachment difficulties (Shaw & Gross, 2008). This contention is borne out by evidence from trials that have tested interventions with parents of young children or with expectant parents (Olds, Sadler, & Kitzman, 2007), epidemiological work highlighting the importance of the early years of development in setting the trajectory for adult outcomes (Shonkoff, Boyce, & McEwan, 2009) and health economic studies demonstrating the cost returns achieved by effective early intervention (Heckman, 2006).

A well-studied example is childhood behavioural problems. These are common, affecting approximately 5% of children (Moffitt & Scott, 2008; Scott, 2008). They have long-term significance, as children with established behavioural problems have much worse outcomes across a wide range of domains of functioning in adolescence and adult life. These outcomes include the following: an increased risk of psychiatric disorders; antisocial behaviour and criminality; drug and alcohol misuse; physical ill health and relationship breakdown (Miner & Clarke-Stewart, 2008; Moffitt, & Scott, 2008; Petitclerc & Tremblay, 2009; Shaw, Gilliom, Ingoldsby, & Nagin, 2003). As well as the considerable unhappiness and morbidity experienced by the young person and their family, enormous costs are also incurred by society through the health, social care and criminal justice systems (Campbell, 1995; Scott, Knapp, Henderson, & Maughan, 2001).

One important conclusion arising from this early intervention work is that parenting emerges as a key risk factor (Moffitt & Scott, 2008; Petitclerc & Tremblay, 2009). Critically, parenting capacities are also modifiable by intervention (Bakermans-Kranenburg, van Ijzendoorn, & Juffer, 2003; Gardner, Dishion, Shaw, & Burton, 2007). However, one feature that has been common to most attempts at early preventive intervention is that they have almost all focused exclusively on intervening with *mothers* and infants, with little attention paid to the role of fathers. There are understandable reasons for this maternal focus; mothers often provide the majority of care for young children, and they are more often available to participate in research. Nonetheless, there is a significant body of research pointing to an important role of fathers in their children’s development across a range of domains, including behavioural development. There are three key bodies of research evidence which suggest that an increased focus on fathers in the prevention of early behavioural problems may be particularly beneficial.

Firstly, although the majority of research addressing parental influence in the early years of children’s lives focuses on mothers, that which has addressed fathers suggests that they may contribute uniquely to children’s development (DeKlyen, Biernbaum, Speltz, & Greenberg,1998; Lamb, 2004; Tamis-LeMonda, Shannon, Cabrera, & Lamb, 2004). Evidence points towards this being particularly the case for behavioural problems (Ramchandani & Psychogiou, 2009). Paternal sensitivity in interactions with their young children predicts better behavioural and psychological outcomes for children later in development (Grossman et al., 2002). Conversely, lower levels of father involvement predict behavioural problems in children. Recent work (Magill-Evans, Harrison, Benzies, Gierl, & Kimak, 2007) suggests that it is possible to improve fathers’ sensitivity in interactions with their infants.

Secondly, there is accumulating evidence that factors which adversely affect fathers’ ability to engage with their children, such as psychiatric disorder, are independently associated with an increased risk of behavioural problems in their young children, and ultimately an increased risk of psychiatric disorders (Connell & Goodman, 2002; Ramchandani & Psychogiou, 2009; Ramchandani, Stein, Evans, & O’Connor, 2005). The precise mechanisms by which these risks are transmitted are yet to be elucidated, but there is emerging evidence that direct effects on fathers’ engagement with their children (Davis, Davis, Freed, & Clark, 2011; Paulson, Keefe, & Leiferman, 2009), and also adverse effects on the marital relationship (Cummings, Keller, & Davies, 2005), are important.

Thirdly, there is evidence that involving fathers in interventions with their infants leads to increased overall efficacy. In a substantial meta-analytic review it was found that those studies that involved fathers as well as mothers had significantly greater effect sizes than those that did not (*d*=1.05 versus 0.42) (Bakermans-Kranenburg et al., 2003). However, it should be emphasised that this is based on a small number of studies involving fathers and to date there are no substantial randomised controlled trials directly comparing an intervention involving fathers with a similar intervention with mothers. In a recent case report, Fletcher (2009) describes the use of Systematic Training in Effective & Enjoyable Parenting (STEEP), a video-feedback intervention, with the father of an infant whose mother had postnatal depression. Its impact was reported to be positive for the father–infant and mother–infant relationships and, unexpectedly, the father–mother relationship.

Three broad approaches have been used in preventive interventions involving parents of young children; attachment theory based, risk factor parent-supporting approaches and social learning theory based (Scott, 2008). All of these approaches have principally focused on mothers. Here we report on the use of one programme, video feedback (Video-feedback Intervention to promote Positive Parenting – VIPP) to engage and work with fathers in an attempt to improve their parenting capacities, and thereby improve their children’s outcomes. This is a preliminary step to using an intervention with both mothers and fathers in a family. VIPP incorporates both attachment and social learning perspectives, and specifically targets parenting capacities that are implicated in the aetiology of a range of childhood problems, including behavioural problems. It has been thoroughly evaluated in five randomised controlled trials with mothers and infants (Juffer, Bakermans-Kranenburg, & van IJzendoorn, 2005; Kalinauskiene et al., 2009; Klein Velderman, Bakermans-Kranenburg, Juffer, & van IJzendoorn, 2006; Stein et al., 2006; van Zeijl et al., 2006). It is a brief (four to six session), home-based intervention (both features that are likely to enhance the participation and engagement of fathers). In addition, it focuses principally on positive aspects of parent–child interaction and builds on them. This is different from many problem-focused approaches, a critical feature with fathers, who are more likely to feel ill at ease with their skills interacting with their infants. The focus is directly on parent–infant interaction through the use of video, so providing direct access to the quality of interaction for each particular parent–infant dyad. As fathers have different patterns of interaction with young children, this allows for the individual tailoring of the treatment in each case, rather than relying on films of other father–infant pairs to demonstrate parenting strategies. Finally, it focuses specifically on changing the parenting processes that are thought to be causally implicated in the aetiology of childhood behavioural problems.

In this study, we set out to use this established early parent–infant intervention (VIPP) with fathers. The purpose of the study was to assess the feasibility (that is, acceptability and deliverability) of the intervention, before it can be used in a formal test of effectiveness in a randomised controlled trial. The aim of the intervention was to enhance fathers’ sensitivity and responsiveness in their interactions with their infants.

## Methods

### Participants and procedure

Consecutive fathers with new young children were approached from an existing longitudinal study of parents and children. Five of eight approached agreed to participate.

The principal outcome of interest was detailed feedback from fathers regarding the acceptability of the intervention. This was collected using an adapted questionnaire measure completed by fathers following the conclusion of the intervention. The secondary outcome was improvement in fathers’ parenting capacities. Thus, at pre- and post-intervention we measured fathers’ engagement in activities with their children, paternal mood and infant temperament.

### Intervention

VIPP is an early preventive intervention used in the first two years of life. It incorporates both social learning and attachment perspectives, and it specifically targets parenting capacities that are implicated in the aetiology of behavioural problems. VIPP was originally developed by the attachment research group at Leiden University in The Netherlands (Juffer, Bakermans-Kranenburg, & van IJzendoorn, 2008), but has now been adapted and used in a number of other countries, including the UK (Stein et al., 2006) and Lithuania (Kalinauskiene et al., 2009). Adaptations of the original VIPP include VIPP-R, an intervention with an additional focus on mothers’ mental representations of attachment, and VIPP-SD, an intervention with an additional focus on sensitive discipline. This has been used with mothers with children aged 1–3 years. VIPP and the adaptations have been thoroughly evaluated in five randomised controlled trials with mothers and their children, showing consistent improvements in maternal sensitivity.

It has a number of key features that make it potentially suitable for use with fathers as well as mothers. These include the following.

- It is a brief and home-based intervention, both features that are likely to enhance the engagement and participation of fathers.
- It focuses principally on positive aspects of parent–child interaction; building on the strengths of each parent. This is different from many problem-focused approaches, a critical feature with fathers, who are more likely to feel ill at ease with their skills interacting with their infants.
- The focus is directly on parent–infant interaction through the use of video, so holds up ‘a mirror of their own daily interactions with their child’ (Juffer et al., 2008, p. 194) and thereby provides direct access to the quality of interaction for each particular parent. As fathers have different patterns of interaction with young children, this allows for the individual tailoring of the treatment in each case, rather than relying on videos of other father–infant pairs to demonstrate parenting strategies (Lambermon & van IJzendoorn, 1989). It also allows for the focus to be on aspects of father–infant interaction that may be different from mother–infant interaction; hence, the aim is not to make fathers more like mothers, but to improve important aspects of father–infant interaction, such as cognitive stimulation and use of warmth in care-giving.

Video feedback treatment (VIPP) is derived from an understanding of attachment theory (Bowlby, 1969), whereby the promotion of sensitive parenting leads to improvement in the core relationship that children have with their primary care-giver. It comprises four sessions that aim to enhance the parent’s capacity to treat the child as a psychological agent by using a number of specific techniques to help the parent consider the infant’s perspective. For this, a range of different care-giving situations are observed and video-recorded, including those that focus on attachment issues. The therapist works with the parent to think about the meaning of the child’s communication shown in the video-recorded clips, and offers different perspectives, not in a didactic form, but by wondering with the parent about the infant’s communication and by using the ‘speaking for the child’ technique (Carter, Osofsky, & Hann, 1991), which helps to verbalise possible interpretations of the infant’s communication. Furthermore, the therapist can help the parent identify the child’s exploratory behaviour and attachment cues and to respond to them appropriately (Juffer et al., 2008). With fathers, watching the video clips, discussing the child’s communication and identifying exploratory behaviour was called ‘Play & Watch’.

The additional components to VIPP, with a focus on sensitive discipline, were not included in this pilot study of the feasibility of a using a video-feedback intervention with fathers. We elected to maintain focus on assessing the feasibility of addressing sensitivity and responsiveness of fathers at this stage.

No specific changes were made to the intervention for the purposes of using it with fathers, with the exception of allowing increased flexibility over the timing of sessions (there were a large number of sessions held before or after typical work hours, and at weekends), and also the place of delivery (although sessions were usually held at home, in one case the feedback sessions were often held at the father’s place of work).

### Measures

#### Detailed questionnaire assessment of therapy acceptability

The acceptability of VIPP was assessed from fathers’ perspective by asking them to provide detailed feedback about the intervention. This was obtained using a self-completion questionnaire that was designed for this study, but based closely on those used in other related pilot work. Quantitative items sought responses on a five-point Likert scale to questions regarding the effect of the intervention on a father’s insight into various aspects of his baby’s mental and social functioning (e.g. understanding, communication, thoughts and feelings). In addition, they were asked about how useful the various components of the intervention were (e.g. watching and reviewing videoed clips of interactions, session location and frequency), and also whether alterations to the intervention would have been acceptable (e.g. involvement of mothers in sessions, recruitment via healthcare service). Qualitative responses were sought on issues including fathers’ expectations of the intervention, their feelings about the ‘Play & Watch’ component, problems identified and general reflections.

#### Paternal mood

This was assessed before and after intervention with the widely used and established Edinburgh Postnatal Depression Scale (EPDS; Cox, Holden, & Sagovsky, 1987). This 10-item scale has been used extensively with mothers, but has also been used with fathers in the postnatal period, and in two separate studies has been shown to demonstrate good sensitivity and specificity in predicting Depressive Disorder (Edmondson, Psychogiou, Vlachos, Netsi, & Ramchandani, 2010; Matthey, Barnett, Kavanagh, & Howie, 2001).

#### Infant temperament

The Infant Characteristics Questionnaire (ICQ) is a factor-analytic screening questionnaire developed by Bates (Bates, Freeland, & Lounsbury, 1979) to measure infant difficult temperament using four subscales. This study used only one of the scales on the questionnaire, the *fussy-difficult* factor. This factor consists of nine questions and is a Likert-based scale with scores ranging from 1 to 7. A higher score on the questionnaire indicates a more difficult or demanding infant. The total score is calculated by adding each question’s score. Fathers completed the ICQ prior to the first ‘Play & Watch’ session, and again after the final session.

#### Father involvement

A 19-item questionnaire measure was used to assess fathers’ involvement with their children. This had previously been used in the Early Childhood Longitudinal Study (Nord et al., 2004) and is described in Bronte-Tinkew, Carrano, Horowitz, and Kinukawa (2008). We used the same five subscales as derived from that original study. These subscales were cognitive stimulation, physical care, paternal warmth, nurturing activities and care-giving activities.

Thus, in all domains (acceptability, paternal mood, infant temperament and father involvement), only self-report measures were used.

### Ethical approval

Ethical approval was obtained from Oxford Research Ethics Committee (REC ref: 08/H0606/78). All fathers provided written informed consent.

## Results

All five fathers completed all of the intervention sessions. There were two boys and three girls in the study; all the children had siblings. At the first session, the children’s ages ranged from six months (the four youngest being born within six weeks of each other) to 15 months. Fathers’ ages ranged from 36 to 53 years. All fathers lived at home with the respective infant’s mother. The three fathers who declined to participate offered brief explanations for this choice. Two reported a lack of interest in participating in research, and the third that he did not have enough time to spare.

Sessions predominantly took place in the evenings or at the weekend. A few sessions were held early in the morning prior to normal working hours. Four fathers had all sessions at their home, and one father had some of the feedback sessions at his place of work and the rest of the sessions at home.

### Fathers’ feedback on the intervention

Fathers, on average, rated the intervention as having had a significant impact on their understanding of their baby’s thoughts and feelings (mean rating 4 = ‘a lot’). Fathers also rated the intervention as having had an impact on their relationship with their baby, their understanding of their baby and their communication with their baby. The mean feedback scores for these three aspects were all 3.6 (between ‘moderate effect’ and ‘a lot’).

Fathers rated the feedback sessions as helpful, scoring on average 3.6 (between ‘moderately helpful’ and ‘a lot’). Individual detailed feedback from fathers included that this was ‘eye opening…overall very interesting’, ‘fascinating’, and ‘great to see’. One father noted that, despite being fascinating, he was ‘not sure if this altered interactions’.

Regarding organisational aspects of the intervention, fathers reported that arranging the sessions around their baby’s schedules posed no problems (due to evening and weekend sessions being available); and that it was, on average, ‘very helpful’ to have sessions at home. One father suggested that it might be difficult if other children were present at home, and that they would require looking after during sessions. Two fathers reported that they would have taken part if sessions were not at home (i.e. in a hospital or clinic), two that they would not have done and one that this would have been dependent on time.

Regarding frequency of sessions (usually held approximately weekly), three fathers reported that this was ‘about right’, and two that sessions were ‘too frequent’. Both fathers who reported that sessions were ‘too frequent’ reported that there were ‘not enough’ visits, whereas the other three fathers reported that the number of visits was ‘about right’.

Fathers were also asked to report on the possible involvement of partners (mothers) in sessions. Mothers were not asked to report on possible joint involvement. Three fathers reported that they would prefer a mix of some sessions together and some separately, and two fathers reported no preference regarding separation or combination. One father commented that it ‘could be great to do both, but could see it as a little intimidating for an unconfident father – as mum is potentially with kids more of the time and might inhibit dad’s natural interactions’.

Fathers were asked to comment on the best features of participation. Comments included that they had been able to ‘see the baby in a new way…and increase understanding of development’, and that they had been able to see features of their interactions with their children in the video playback sessions that they had not identified in real time. The only negative feature of participation reported, from one father, was the time involved.

### Other questionnaire measures

This was a pilot study to assess the acceptability and deliverability, not the effectiveness, of the intervention. On this basis, and the small size of the sample, formal statistical tests were not conducted on the data yielded from the questionnaire measures. Pre- and post-treatment mean scores are shown in Table 1.

**Table 1.** Fathers’ scores at pre- and post-intervention.

Measure	Pre-treatment mean (SD)	Post-treatment mean (SD)
EPDS	3.2 (4.5)	2.6 (5.3)
ICQ	25 (5.6)	23.8 (5.2)
Father involvement		
*Cognitive stimulation*	4.0 (2.4)	4.6 (1.7)
*Care-giving*	12.8 (1.9)	11.6 (1.8)
*Warmth*	9.6 (0.5)	9.8 (0.4)
*Nurturing*	4.8 (2.5)	6 (3.4)
*Monitoring/ supervision*	10.6 (2.1)	10 (0.7)

*Note*. EPDS: Edinburgh Postnatal Depression Scale, ICQ: Infant Characteristics Questionnaire

Both depression scores and fathers’ ratings of their infants’ difficult temperament reduced slightly after treatment (changes of 0.6 and 1.2, respectively). The picture on the degree of father involvement was more mixed, with fathers describing marginally more cognitive stimulation, warmth and nurturing in their interactions, but less care-giving and supervision. This is in line with the selected targets of the intervention – to improve paternal sensitivity and responsiveness (here measured as ‘cognitive stimulation’, ‘warmth’ and ‘nurturing’).

## Discussion

This pilot study of a video-feedback intervention (VIPP) to improve interactions of fathers with their infants focused on developing an acceptable and deliverable intervention. All five fathers completed all of the intervention sessions. Fathers’ feedback on the content of sessions was generally positive, and some specified that the video-feedback (‘Play & Watch’) component of the intervention had improved their understanding of their infants’ behaviour and development. Fathers’ feedback also suggests that flexibility, both to conduct sessions outside typical working hours and in the home environment, is fundamental to successful delivery.

To our knowledge, this is the first study to report on the use of VIPP with fathers. There are several limitations to this pilot study, including the modest sample size, the use of fathers from a non-clinical population, and the lack of a control group or observational measures. In light of these limitations, the results might not be generalisable to fathers in a clinical setting, and do not allow us to comment on the transferability of results to fathers experiencing psychological distress, or to fathers of infants at risk of adverse developmental outcome.

Nonetheless, the results of this pilot study are encouraging. They provide a basis on which to develop the VIPP intervention for fathers, and then to conduct more robust evaluations of it, with larger sample sizes, in order to provide more robust estimates of its effectiveness.

On the basis of this pilot, where most fathers reported that they would prefer a mix of sessions in conjunction with mothers, future studies could augment VIPP for clinically identified mothers by including fathers more actively in order to assess both its effectiveness and, with appropriate design, to test putative mechanisms of the effects of including fathers. However, the opinions of mothers regarding joint sessions should also be sought to assist with planning and designing such an intervention.

The three fathers who chose not to participate reported that they lacked time or interest in research participation. This lack of interest is a challenge to be met in future studies of interventions for fathers.

The current study is also useful in considering the development of interventions for fathers with identified risk factors for adverse child development, for example, depression in the postnatal period (Ramchandani et al., 2005). Fathers with depression in the postnatal period demonstrate impairments in their interactions with their offspring, even from the first few months of a child’s life (Sethna, Murray, & Ramchandani, in press), and VIPP is a promising candidate intervention to address this. Future studies might consider the adaptation of VIPP for use with fathers from clinical populations. Given the different characteristics of clinical fathers, it is likely that such an adaptation would itself benefit from development in a pilot study.

Further key considerations in future studies of interventions to promote fathers’ sensitivity are the possible gender-specific effects that characterise paternal sensitivity (in distinction to those of interactions that characterise maternal sensitivity), with mothers appearing to be more reliable sources of comforting interactions and fathers as preferred partners in playful interactions (Clarke-Stewart, 1978; Lamb, 1977, 2004). If interventions developed to enhance sensitive parenting in mothers are to be used most effectively with fathers, the behavioural targets might be revised to include those shown to be more characteristic of paternal sensitivity in father–infant interactions. Specifically, VIPP might focus on enhancing fathers’ sensitivity by including more stimulating play tasks and via greater emphasis on modifying their sensitive behaviours in this mode of interaction.

Recent research has considered possible neurobiological bases for the different interaction styles between mothers and fathers. Research with mammals has implicated the neuropeptide oxytocin (OT) in parent–infant bonding (e.g. Carter, 1998). Recent research that has examined the role of OT levels in human parent–infant interaction has found that mothers’ and fathers’ OT levels are associated with different modes of interaction with infants and children; in mothers OT is associated with affectionate behaviour, in fathers with stimulatory play (Feldman, Gordon, Schneiderman, Weisman, & Zagoory-Sharon, 2010; Gordon, Zagoory-Sharon, Leckman, & Feldman, 2010). The causal role of OT on fathers’ parenting behaviour was investigated by Naber, van IJzendoorn, Deschamps, van Engeland, and Bakermans-Kranenburg (2010), who showed that OT administered intranasally to fathers caused increased stimulating play with their children (aged between 18 months and 5 years). This finding raises the possibility of investigating the differential and cumulative effects of combining a psychological intervention to enhance paternal sensitivity (VIPP) with a novel pharmaceutical treatment – intranasal administration of OT.

In light of the demonstrated links between insensitive parenting, insecure infant attachment and adverse child development, there is a need for acceptable and effective interventions early in life to provide both primary preventive opportunities and effective intervention where difficulties exist. VIPP provides a potentially positive and sustainable intervention that could be used in these circumstances.
